# Supplementary material for: Content-rich biological network constructed by mining PubMed abstracts
Source: BMC Bioinformatics. 2004 Oct 8;5:147. doi: 10.1186/1471-2105-5-147 (PMC528731; doi:10.1186/1471-2105-5-147)
Supplement: Additional File 2 — The original results of the above study (non-essential files are deleted to keep the file size under the limit set by BMC bioinformatics). [file 1471-2105-5-147-S2.bz2 › chilibotAdditionalFile2/dip20/443ID9169459E10419/html/CCL20_CCR6.html]

 


 **CCL20** and **CCR6** 
  
Found 51 abstracts in PubMed, retrieved 20.  
 

 What does Google say? 
 PDF only 
| .edu only 

---

**Interactive relationship** (e.g. stimulation, inhibition, etc)

**Neutral relationship**- MIP 3alpha  **CCL20**  is the unique chemokine ligand for  **CCR6** , a receptor with a restricted distribution.  Ref: 12760962 Am J Respir Cell Mol Biol< MedlineTA>Am J Respir Cell Mol Biol,
- **CCR6**  is the receptor for the chemokine MIP 3 alpha  [ **CCL20** ]   **CCL20** .  Ref: 12081481 Biochemistry, 2002
- Macrophage inflammatory protein 3alpha  **CCL20**  is a recently identified chemokine that binds to  **CCR6**  and acts as a chemoattractant for memory differentiated T cells, B cells, and immature dendritic cells.  Ref: 12528183 Glia, 2003
- A close association was observed between  **CCL20**  producing cells and CD1a cells, suggesting the contribution of  **CCL20**  to  **CCR6**  cell homing.  Ref: 11994492 J Immunol, 2002
- Only on follicular B cells the cognate receptor for  **CCL20** , CC chemokine receptor 6  [ **CCR6** ]   **CCR6** , was expressed strongly and a functional transmigration assay with  **CCR6**  negative B cells demonstrated conclusively the sole signaling of  **CCL20**  through  **CCR6** .  Ref: 12816871 Blood< MedlineTA>Blood, 2002
- **CCL20**  is a CC type chemokine that binds to and activates CC chemokine receptor 6  [ **CCR6** ]   **CCR6** .  Ref: 12149255 J Biol Chem, 2002
- **CCR6**  is the only known receptor for the chemokine macrophage inflammatory protein MIP 3alpha CC chemokine ligand CCL 20  [ **CCL20** ] .  Ref: 11994436 J Immunol, 2002
- Preincubation with the  **CCR6**  ligand  **CCL20**  induced liver sorted tumour cells to preferentially colonize the liver, demonstrating an association between liver metastasis and  **CCR6**  expression in the mouse.  Ref: 12791091 Scand J Immunol, 2003
- The generality of the latter finding was demonstrated by the fact that  **CCL20**   **CCR6**  interactions increase the basal proliferation rate of CD62L lo CD4 T cells irrespective of their thymic in non OM transgenic mice or extrathymic in LckOM mice origin.  Ref: 12702501 Blood< MedlineTA>Blood, 2003
- Chemokine ligand 20  [ **CCL20** ]   **CCL20**  and human beta defensins HBDs share structural and functional properties, including anti parallel beta pleated sheet core structures, charge distribution, and signaling to adaptive immune cells via the highly selective  **CCR6**  receptor.  Ref: 12748059 Am J Respir Cell Mol Biol< MedlineTA>Am J Respir Cell Mol Biol, 2003
- In contrast to the starting JK lines, the resulting cell line JK fucosyltransferase VII  **CCR6**  migrated 6 fold better to  **CCL20**  in chemotaxis assays, arrested in response to  **CCL20**  that was immobilized to plastic, and demonstrated a 2.5 fold increase in adhesion to activated HDMEC p = 0.001.  Ref: 12193700 J Immunol, 2002
- Of particular interest is the fact that the enhanced activity on B cells was not associated with an increase in  **CCR6**  expression as assessed by levels of receptor mRNA, surface staining, or MIP 3alpha  **CCL20**  binding sites, or by a change in the affinity of the receptor for ligand.  Ref: 11994436 J Immunol, 2002

**Non-interactive relationship** (e.g. studied together, co-existance, homology, etc.)

- Furthermore, regardless of the tissue site, LCH lesions markedly overexpressed  **CCL20**  MIP 3alpha, the ligand for  **CCR6** .  Ref: 12743170 J Exp Med, 2003
- Desensitization of  **CCR6**  on naive B cells with  **CCL20**  resulted in receptor downregulation and reduced B cell adhesion.  Ref: 12816871 Blood< MedlineTA>Blood, 2003
- We then focused on the expression of  **CCL20**   **CCR6**  and CCL19 CCR7, CCL21 CCR7 chemokine receptor complex, which control immature and mature DC migration respectively.  Ref: 11994492 J Immunol, 2002
- Activated cells are thought to down regulate  **CCR6** , whose ligand macrophage inflammatory protein 3 alpha  [ **CCL20** ]  MIP 3 alpha  [ **CCL20** ]   **CCL20**  is expressed in skin, and up regulate CCR7, whose ligands are in lymphoid tissues.  Ref: 12642342 Blood, 2003
- The chemokine receptor  **CCR6**  is unusual in that it mediates leukocyte chemotaxis in response to a single chemokine,  **CCL20**  macrophage inhibitory factor 3alpha, as well as in response to a family of antimicrobial peptides termed beta defensins.  Ref: 12514792 Hum Pathol, 2002
- Dual label confocal microscopy revealed that LFA 1 CD18 and CD11a, but not CXCR4, colocalized with clustered  **CCR6**  in the presence of immobilized  **CCL20** .  Ref: 12193700 J Immunol, 2002
- MIP 3alpha  **CCL20**  induces selective migration of DCs because  **CCR6**  is expressed on some immature DCs but not on CD14 DC precursors or mature DCs.  Ref: 12760962 Am J Respir Cell Mol Biol< MedlineTA>Am J Respir Cell Mol Biol, 2002
- Expression of the specific receptor of MIP 3 alpha  [ **CCL20** ] ,  **CCR6** , was also increased in HTLV I infected T cell lines, suggesting an autocrine and or paracrine mechanism to establish the pathogenesis of HTLV I associated diseases.  Ref: 11809734 Int Immunol, 2002
- SIV infection altered the expression of chemokines CCL19 MIP 3beta, CCL21 6Ckine, and  **CCL20**  MIP 3alpha and of chemokine receptors CCR7 and  **CCR6**  that drive DC trafficking.  Ref: 12406887 Blood, 2003
- Because human liver constitutively expresses  **CCL20** , it could attract and select  **CCR6**  cancer cells.  Ref: 12791091 Scand J Immunol, 2003
- We have previously reported that  **CCL20**  and  **CCR6**  mRNAs are expressed in the CNS of SJL mice with experimental autoimmune encephalomyelitis EAE.  Ref: 12528183 Glia, 2003
- Human B cells become highly responsive to macrophage inflammatory protein 3 alpha  [ **CCL20** ]  CC chemokine ligand 20  [ **CCL20** ]  after cellular activation without changes in  **CCR6**  expression or ligand binding.  Ref: 11994436 J Immunol, 2002
- CCL19 MIP 3beta,  **CCL20**  MIP 3alpha,  **CCR6** , and CCR7 expression increased in lymph nodes during the early systemic burst of viral replication acute infection.  Ref: 12406887 Blood, 2003
- In addition, 3 6 cell lines migrated in response to MIP 3 alpha  [ **CCL20** ]  and IP 10, ligands for  **CCR6**  and CXCR3, respectively.  Ref: 12529679 Leukemia, 2003
